# Non-invasive monitoring of cerebral blood flow during cardiac surgery for congenital heart disease - transfontanellar Doppler as additional tool

**DOI:** 10.1038/s41390-024-03576-8

**Published:** 2024-10-14

**Authors:** Matthias Gorenflo

**Affiliations:** University Hospital Heidelberg, Clinic of Paediatric & Congenital Cardiology, Heidelberg, Germany

Children with congenital heart disease are at risk for impaired cerebral perfusion during cardiopulmonary bypass surgery.^1^ Disturbances in cerebral flow during cardiopulmonary bypass as well as circulatory arrest may lead to neurological injury in patients that already in utero are exposed to altered perfusion by structural heart disease as depicted by Barkhuizen et al.^2^ Monitoring of cerebral perfusion during cardiopulmonary bypass is therefore of utmost importance. In an ideal world the team performing cardiac surgery in critically ill neonate would have precise data on overall and regional cerebral perfusion at any single moment of the procedure. Current techniques such as near-infrared spectroscopy (NIRS) provides an estimate of regional cerebral tissue oxygenation as a surrogate parameter of cerebral perfusion. NIRS is subject to many errors such as limited penetration.^3^

Leth-Olsen and colleagues present a pilot study using serial transfontanellar ultrasound Doppler measurements of blood flow velocities during CPB (Leth-Olsen study).^4^ Data obtained with the Doppler technique correlated well to haemodynamic events during the operation. Post-processing of Doppler data demonstrated that autoregulation of cerebral is impaired in a large proportion of time while on cardiopulmonary bypass.

Doppler technique per se is prone to various sources of error such as insufficient spatial resolution, measurements in a non circular vessel and asymmetrical profile or angle estimation.^5^

It is always to remember that Doppler technique will obtain data for blood flow *velocity* but not for blood flow (in l/min) and tissue perfusion. The authors (Leth-Olsen study) have highlighted the importance of applying various techniques (such as NIRS) in combination with transfontanellar ultrasound Doppler measurements simultaneously in a given patient in order to increase the likelihood to early detect problems with cerebral perfusion during cardiopulmonary bypass in neonates and infants with congenital heart disease.^4^

At present, the problems associated with monitoring of cerebral perfusion should present a stimulus to increase further technical innovation and studies that relate such measurements to clinical outcome. The data provided by Leth-Olson and co-workers should stimulate further research in larger cohorts in order to determine the potential advantages but also the limitations of such methods.
